# Gab1 Is Modulated by Chronic Hypoxia in Children with Cyanotic Congenital Heart Defect and Its Overexpression Reduces Apoptosis in Rat Neonatal Cardiomyocytes

**DOI:** 10.1155/2015/718492

**Published:** 2015-05-19

**Authors:** Myriam Cherif, Massimo Caputo, Yoshikazu Nakaoka, Gianni D. Angelini, Mohamed T. Ghorbel

**Affiliations:** ^1^Bristol Heart Institute, School of Clinical Sciences, University of Bristol, Research Level 7, Bristol Royal Infirmary, Upper Maudlin Street, Bristol BS2 8HW, UK; ^2^RUSH University Medical Center, Chicago, IL 60612, USA; ^3^Department of Cardiovascular Medicine, Osaka University Graduate School of Medicine, 2-2 Yamadaoka, Suita, Osaka 565-0871, Japan

## Abstract

Gab1 (Grb2 associated binding protein 1) is a member of the scaffolding/docking proteins (Gab1, Gab2, and Gab3). It is required for fibroblast cell survival and maintaining cardiac function. Very little is known about human Gab1 expression in response to chronic hypoxia. The present study examined the hypothesis that hypoxia regulates Gab1 expression in human paediatric myocardium and cultured rat cardiomyocytes. Here we showed that Gab1 is expressed in myocardial tissue in acyanotic and cyanotic children with congenital heart defects. Gab1 protein was upregulated in cyanotic compared to acyanotic hearts suggesting that Gab1 upregulation is a component of the survival program initiated by hypoxia in cyanotic children. The expression of other Gab1 interacting partners was not affected by hypoxia and Gab1 regulation. Additionally, using an *in vitro* model, we demonstrated that overexpressing Gab1 in neonatal cardiomyocytes, under hypoxic condition, resulted in the reduction of apoptosis suggesting a role for this protein in cardiomyocyte survival. Altogether, our data provide strong evidence that Gab1 is important for heart cell survival following hypoxic stress.

## 1. Introduction

Heart malformation during embryonic development can cause congenital heart diseases (CHD). These affect one baby in 125 live births and tetralogy of Fallot (TOF) represents the most common form of the “blue baby syndrome.” In UK, one baby in 3,600 is born with TOF malformation [1]. TOF malformation exhibits four abnormalities. These include a ventricular septal defect (VSD), right ventricular hypertrophy, overriding of the aorta, and pulmonary stenosis (PS) [2]. The causes that induce TOF are not fully understood but the aetiology is thought to be multifactorial. Some studies associated TOF with untreated maternal diabetes, phenylketonuria, and intake of retinoic acid. In addition, chromosomal abnormalities (such as trisomies 21, 18, and 13) have been shown to exhibit a higher TOF incidence [3]. The degree of stenosis varies between individuals with TOF and is the primary determinant of symptoms and severity. Indeed, TOF is divided into two categories: acyanotic (pink) and cyanotic (blue), depending on the blood oxygen saturation. Although successful corrective surgery of heart defects exists, there is an increased risk of morbidity and mortality in cyanotic children compared with acyanotic [4]. There is evidence that an unintended reoxygenation injury occurs in myocardium of cyanotic patients due to the delivery of high levels of oxygen during cardiopulmonary bypass (CPB) used in surgery, which does not match preoperative levels of oxygen in these children. Reoxygenation injury produces an increase in free radical production, which may result in cell damage [5].

In previous study, we have shown that chronic hypoxia in pediatric patients with TOF triggered the expression of network of genes associated with apoptosis and reduced the expression of genes involved in myocyte contractility and function [6]. This state of hypoxia in TOF children may be responsible for the susceptibility of cyanotic children to reoxygenation injury during and after surgery. We have also shown that concomitant with the initiation of the injurious program a protective program is triggered by cyanosis. Gab1, shown to significantly increase at messenger level in cyanotic compared to acyanotic patients [6], could be an important player in this protective program.

Grb2 associated binding protein 1 (Gab1) is a member of the scaffolding/docking proteins (Gab1, Gab2, and Gab3) [7, 8]. Gab1 knockout mice are not viable and display impaired development of heart, placenta, skin, and muscle [9]. In cultured cardiomyocytes, Gab1 is shown to interact with tyrosine phosphatase SHP2 and to promote cardiac hypertrophy [10]. There is evidence that Gab1 is essential for cardiac function in the postnatal heart* in vivo* [11]. In addition, Gab1 has been shown to exert an antiapoptotic role in mouse embryonic fibroblasts and is activated through tyrosine phosphorylation following oxidative treatment (H_2_O_2_) [12]. In their investigation, Holgado-Madruga and Wong identified Gab1 as an important component in oxidative stress signalling with an essential role in the activation of c-Jun NH(2)-terminal kinase (JNK) and the influencing of cell survival [12]. This Gab1 antiapoptotic role in fibroblasts following oxidative treatment [12] has led us to hypothesise that Gab1 may play similar role in cardiac tissue and cardiac myocytes subjected to hypoxia.

In this study, we investigated the effects of cyanosis on Gab1 in myocardium samples from paediatric patients suffering from TOF and we examined the effects of hypoxia in primary cultures of rat neonatal cardiomyocytes on Gab1 and its possible role in cell survival.

## 2. Materials and Methods

### 2.1. Reagents

All reagents were from Sigma (UK) except those stated otherwise. Gab1 antibody was from Millipore. Antibodies against SHP2 and p85 were from Cell Signalling Technology (UK). GAPDH antibody was from Research Diagnostics Inc. (UK).

### 2.2. Cardiac Biopsies

The collection of human right ventricle specimens used in this study was approved by the North Somerset and South Bristol Research Ethics Committee (REC reference 07/H0106/172), the National Research Ethics Service, England. Parental informed written consent was gained for all patients. Patients with a diagnosis of cyanotic (O_2_ saturation 79.6 ± 7.5%; age 10.6 ± 5.5 months) or acyanotic (O_2_ saturation 94.2 ± 3.5%; age 9.5 ± 2.3 months) tetralogy of Fallot undergoing surgical repair at the Bristol Royal Hospital for Children were studied.

Five ventricular biopsy specimens per group were collected from the right ventricle of acyanotic and cyanotic TOF patients by using “True-Cut” needle immediately after institution of cardiopulmonary bypass (CPB). Each specimen was immediately put in liquid nitrogen for protein extraction.

### 2.3. Immunohistochemistry

Right ventricular specimens were fixed in 4% paraformaldehyde, washed in PBS, and embedded in paraffin and 4 *μ*m sections were obtained. Immunohistochemistry was performed using the ABC-Kit from Dakocytomation. Slides were observed with an Olympus B40 microscope. Pictures were taken using a Media Cybernetics camera (Bethesda, MD, USA) and analysed with proimage plus software (Bethesda, MD, USA).

### 2.4. Rat Neonatal Primary Culture

All the procedures involving laboratory animals conformed to the guidelines and regulations of the University of Bristol and the United Kingdom Home Office. Neonatal rat cardiomyocytes were cultured as previously described with minor modifications [13, 14]. One cell culture preparation was used for each experiment and each experiment was repeated three times using different cell cultures prepared at different time from pooled neonatal rat hearts. For each cell culture, 14–24 neonatal rats were used to harvest hearts. Hearts were quickly removed from one- to three-day-old Wistar rats and only ventricles were kept. They were washed with PBS three times and incubated with 0.05% trypsin and 0.02% EDTA for 30 minutes. They were then enzymatically digested six times for fifteen minutes in 0.1% trypsin and 0.02% EDTA in PBS. Digestion was stopped by addition of foetal calf serum at a final concentration of 30%. Cells were then centrifuged at 400 g for five minutes and resuspended in DMEM supplemented with 10% FBS and 1% P/S. Cells were then incubated one to two hours in a T75 flask to allow noncardiac myocytes (mainly cardiac fibroblasts) to adhere to plastic. They were then plated in gelatin-coated plates at a density of 1.28 × 10^5^ cells per cm^2^. After 40 hours of culture, the medium was changed in DMEM supplemented with 20% M-199 and 1% P/S. All treatments were performed on three-day-old cultures. Hypoxia was induced by placing the cells in a hypoxic chamber where oxygen levels could be monitored (Biospherix ProOx C21, Lacona, NY, USA). The ProOx is connected to a sensor, which monitors the oxygen concentration within the host chamber. The ProOx quickly infuses nitrogen (hypoxia) in the chamber and reaches a set point. In this study, the chamber was set to 0.2% O_2_ and 5% CO_2_ in a humid atmosphere for 24 hours.

### 2.5. Immunocytochemistry

Cells were grown on gelatin coverslips (Nunc, UK) at a density of 1.32 × 10^5^ cells per cm^2^. Immunocytochemistry was performed on normoxic and hypoxic (24 hours, 0.2% O_2_, 5% CO_2_) cells. Cells were washed twice with PBS, fixed with 4% paraformaldehyde for 15 minutes, washed 3 times with PBS, and washed with 0.1% Triton X-100 3 times for 5 minutes. They were then incubated with NH_4_Cl for 10 minutes, washed three times with PBS, and blocked using 5% goat serum (Dakocytomation, Dako, UK) for 45–60 minutes at room temperature. Cells were incubated in Gab1 overnight at 4°C, washed three times, incubated in anti-rabbit conjugated with Alexa-488 (Invitrogen, UK) for 1 hour at room temperature, washed again, and incubated with mouse antisarcomeric actin (Dakocytomation, Dako, UK) and then with anti-mouse conjugated with Texas Red (Vector Laboratories, UK). Cells were then mounted in VECTASHIELD and observed with a Leica AOBS SP2 confocal microscope (MRC Cell Imaging Facility, University of Bristol, UK), using excitation filters at 340–380 nm, 450–490 nm, and 515–560 nm for DAPI, Alexa-488, and Texas Red, respectively. The slides were observed on a 63x lens and pictures were taken using the Leica software (Leica, Bucks, UK).

### 2.6. Use of Adenoviruses

Adenoviruses expressing Gab1 wild type were previously used [10, 15]. Adenovirus expressing wild type Gab1 was referred to as Ad-Gab1-WT. Control adenovirus expressing Ad-*β*-galactosidase was a kind gift from Dr. Steve White (University of Bristol, UK). Cardiomyocytes were infected with adenoviruses diluted in DMEM supplemented with 20% M199, 1% FBS, and 1% P/S for 24 hours. Then, the medium was changed to DMEM supplemented with 20% M199 and 1% P/S. The infected cells were then subjected to normoxia or hypoxia for 24 hours.

### 2.7. Western Blotting

Five myocytes culture dishes (from the same cell preparation) per group (normoxia versus hypoxia) were used in the rat cell culture part. The* in vitro* experiment was repeated three times using new cell preparation each time. Total proteins were extracted from both clinical and rat samples and quantified. For electrophoresis, protein samples were prepared by adding 4x Laemmli buffer (0.24 M Tris pH 6.8, 6% SDS, 40% sucrose, 0.04% bromophenol blue, and 10% *β*-mercaptoethanol), heated to 95°C for 5 min, and loaded on a 8–10% SDS gel. Separated proteins were transferred to Hybond nitrocellulose membrane (Amersham, UK) which was subsequently blocked in 5% nonfat dry milk/TBS-T (TBS-T; 20 mM Tris pH 7.4, 1.37 M NaCl, 1% Tween) for 1 h and incubated in primary antibodies overnight at 4°C. Membranes were washed three times in TBS-T and then incubated in appropriate anti-rabbit or anti-mouse secondary antibody (Amersham, UK) for 1 h at room temperature. Membranes were washed 3 times in TBS-T, antibody bound HRP was detected using ECL (Amersham, UK), and membranes were exposed to Hyperfilm (Amersham, UK). Protein bands were quantified using NIH Image J software. The expression level of each protein was normalized to the loading control, GAPDH, and then the obtained values for experimental (cyanotic or hypoxic) were divided per control value (acyanotic or normoxia). Representative blots were presented in the western blotting figures.

### 2.8. Apoptosis Assay

Cell death was investigated with the “*in situ* cell death detection” kit from Roche. Rat neonatal cardiomyocytes were cultured in 4-well chamber slides (Nunc, UK) at a density of 1.5 × 10^5^ cells per cm^2^. Cells were washed twice with PBS and permeabilized for 1 hour at room temperature with 0.1% Triton (v/v) in 0.1% sodium citrate (v/v). They were then washed three times in PBS and incubated with the labelled enzyme for 1 hour at 37°C. They were washed twice, mounted in VECTASHIELD containing DAPI, and observed with an Olympus B40 microscope. Apoptosis was quantified by counting apoptotic cells in 25 different fields or 400 cells. Pictures were taken using a Media Cybernetics camera (Bethesda, MD, USA) and analysed with proimage plus software (Bethesda, MD, USA).

### 2.9. Statistical Analysis

All data were analyzed using the software Instat 3.1 (GraphPad). Results are expressed as ± standard error of mean (±SEM). Statistical significance was assessed by one-way ANOVA or Student's* t*-test. A value of *P* < 0.05 was considered to be statistically significant.

## 3. Results

### 3.1. Gab1 Protein Is Expressed in Human Heart Sections of Both Cyanotic and Acyanotic Congenital Heart Patients

**Figure 1:**
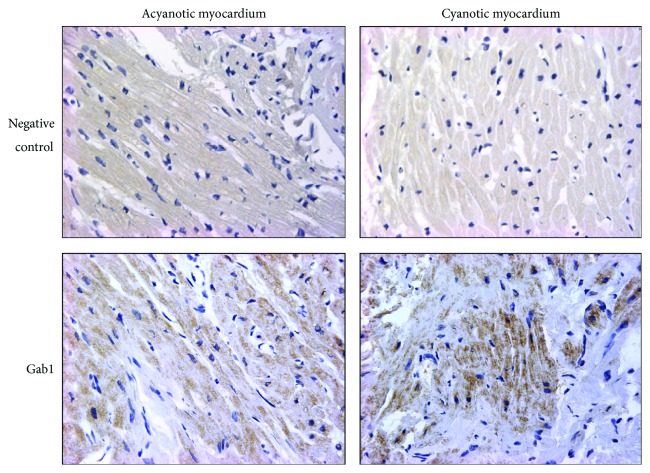
Expression of Gab1 in acyanotic and cyanotic paediatric myocardium tissues. Immunohistochemical analysis of paraffin embedded heart tissue from acyanotic and cyanotic patients using Gab1 specific antibody. Negative controls are section processed without using primary antibody. Magnification: ×200.

The analysis of fixed paediatric heart biopsy sections by immunohistochemistry showed expression of GAB1 in heart tissue taken from both acyanotic and cyanotic patients undergoing corrective surgery for congenital heart defects (Figure 1).

### 3.2. Gab1 Protein Expression Is Upregulated in Cyanotic Patients Compared to Acyanotic

**Figure 2:**
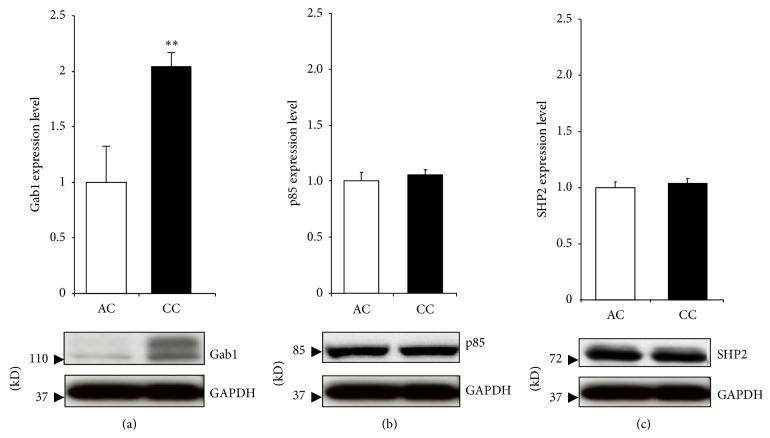
Gab1 protein expression in myocardium of cyanotic (CC) and acyanotic (AC) patients. Myocardium biopsies were homogenized to isolate protein content and western blot analysis performed using Gab1, p85, SHP2, and GAPDH antibodies. All results were normalised to GAPDH levels. Data are mean ± SEM. ^**^ *P* < 0.01 (*n* = 5).

Western blot analysis of proteins extracted from children myocardium biopsies revealed a significant upregulation of Gab1 protein expression in cyanotic patients (Figure 2(a)). Additionally, we investigated the protein expression of Gab binding partners, SHP2 and the regulatory subunit of PI3K (p85). The levels of both Gab binding partners proteins were unaffected (Figure 2(b)).

### 3.3. Gab1 Shows a Cytoplasmic Localization in Neonatal Heart Cardiomyocytes

**Figure 3:**
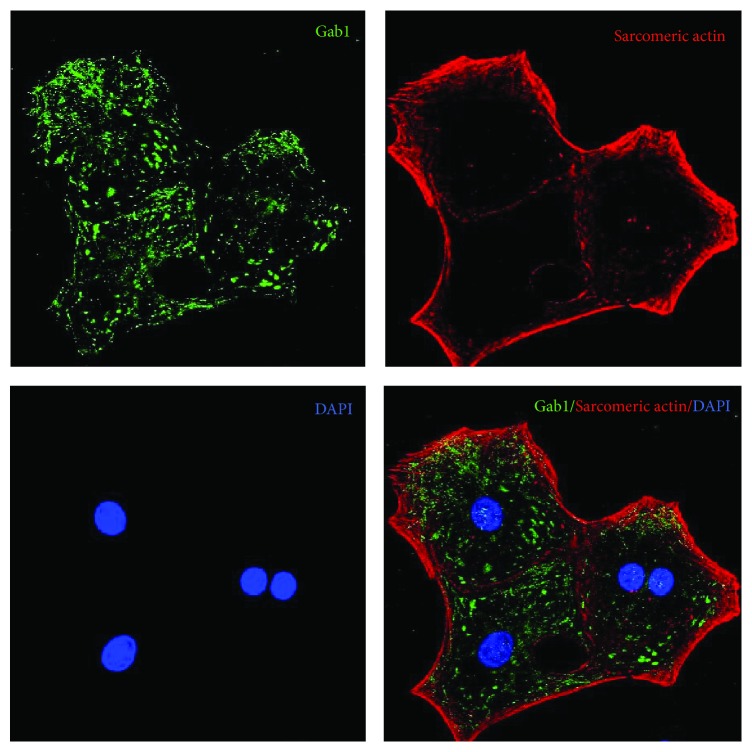
Localization of Gab1 in cultured rat cardiomyocytes. Cells were cultured for 5 days* in vitro* then fixed and stained with specific antibodies. Cardiomyocytes were stained for Gab1 (green) and sarcomeric actin (red) and then counterstained for nuclei with DAPI (blue). Magnification: ×1200.

Gab1 appeared to be expressed in rat neonatal cardiomyocytes (Figure 3). It is interesting to note that Gab1 is located mainly in the cytoplasm of normoxic cells with some nuclear expression.

### 3.4. Hypoxia Produce a Downregulation of GAB1 in Cultured Rat Cardiomyocytes

**Figure 4:**
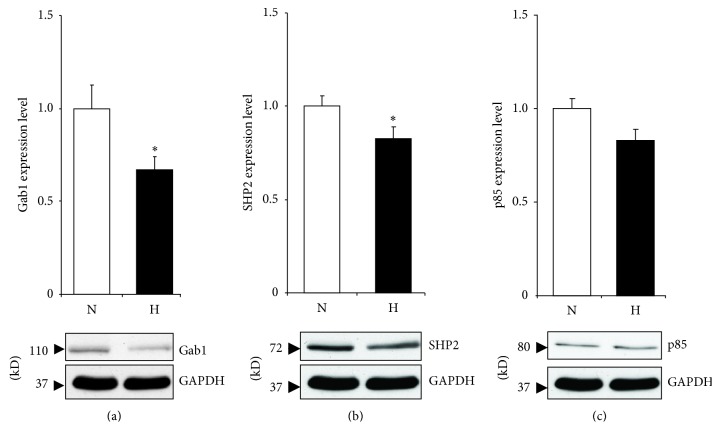
Gab1 protein expression in rat cardiomyocytes following 24 h of hypoxic treatment. Cells were treated with hypoxic conditions (H) by incubating in a chamber with 5% CO_2_/0.2% O_2_ or normoxia (N) by leaving cells in normal CO2 incubator. After 24 h cells were lysed and western blot analysis performed using GAB1 (a), SHP2 (b), and p85 (c) antibodies. All results were normalized to GAPDH levels. Data are mean ± SEM. ^*^ *P* < 0.05 (*n* = 5).

As there is a limit to what can be done using human biopsies, an* in vitro* model of cyanosis would be advantageous. Both GAB1 and SHP2 were downregulated at the protein level following hypoxia when compared to the normoxic control (Figures 4(a) and 4(b)). However, hypoxia did not affect p85 protein expression (Figure 4(c)).

### 3.5. Gab1 Overexpression Reduces Apoptosis during Hypoxia in Rat Cardiomyocytes

**Figure 5:**
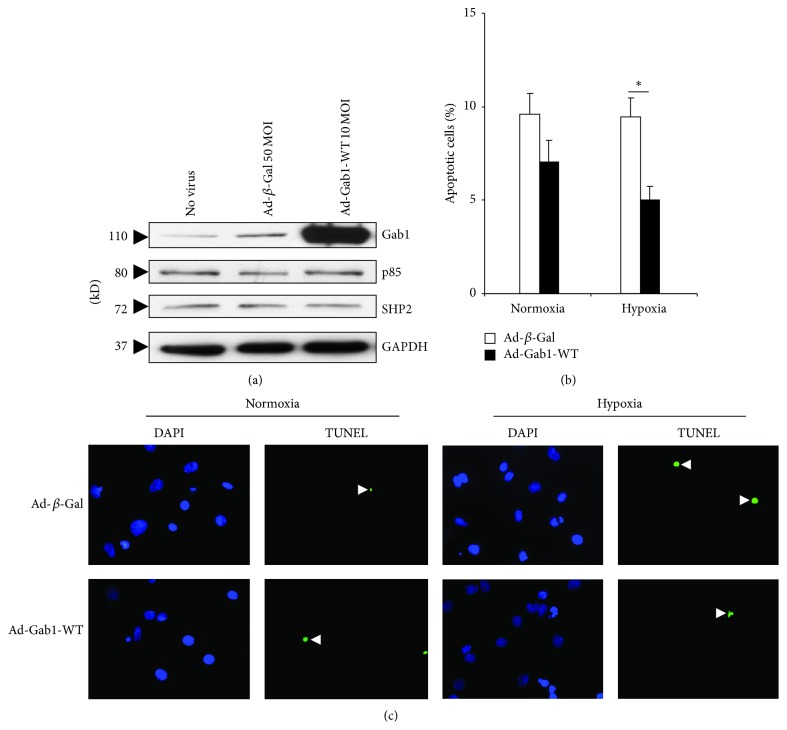
(a) Protein expression level of Gab1, p85, and SHP2 in infected rat neonatal cardiomyocytes. Cardiac myocytes were infected with Ad-*β*-Gal (50 MOI) and Ad-Gab1-WT (10 MOI), for 24 hours. GAPDH was used as a loading control. (b) Apoptosis quantification of infected rat neonatal cardiomyocytes subjected to normoxia and hypoxia. Cardiac myocytes were infected with Ad-*β*-galactosidase (Ad-*β*-Gal) and Ad-Gab1-WT, for 24 hours. Data are mean ± SEM. ^*^ *P* < 0.05. (c) Representative pictures of the TUNEL assay performed on rat neonatal cardiomyocytes infected by Ad-*β*-Gal or Ad-Gab1-WT and subjected to normoxia or hypoxia. Arrows show apoptotic nuclei (green fluorescence). Cell nuclei were stained by DAPI (blue fluorescence). Magnification: ×400.

We first examined the efficiency of cell infection by Ad-Gab1-WT (Figure 5(a)). Infection of rat neonatal cardiomyocytes with Ad-Gab1-WT was successful as demonstrated by the increase of Gab1 protein expression compared to cells infected with Ad-*β*-Gal (Figure 5(a)). In addition, the infection with both viruses did not affect the protein expression of p85, SHP2, and GAPDH.

During normoxia, the overexpression of Gab1 in cardiomyocytes did not alter the percentage of apoptotic cells significantly. However, during hypoxia Gab1 overexpression reduced significantly apoptosis in rat neonatal cardiomyocytes (Figures 5(b) and 5(c)).

## 4. Discussion

Our study revealed a significant upregulation of Gab1 protein expression in cyanotic TOF patients. This result confirmed our previous findings by microarray analysis [6]. The upregulation of Gab1 protein expression in cyanotic patients may suggest an increase of survival signalling mediated through Gab1 in cyanotic patients, independently of SHP2 and p85. These data are the first to implicate Gab1 in cardioprotective signalling in cyanotic patients in response to chronic hypoxia stress.

Gab1 protein levels were downregulated during hypoxia in neonatal rat cardiomyocytes. These are also the first results to implicate Gab1 in the cardiomyocytes response to hypoxia. The difference between the* in vivo* and* in vitro* data regarding the response to hypoxia can be explained by the complexity of the* in vivo* situation in comparison to the relative simplicity of the* in vitro* model. Furthermore, it can be explained by the short time of hypoxia protocol (24 h) used for rat myocytes compared to the patients that stayed cyanotic for months before surgery. Additionally, this may be attributed to the difference between TOF patients' tissue specimens and neonatal rat ventricle. Neonatal rat ventricle includes LV and RV tissues, whereas TOF patients' tissue was only from RV. It is well known that there is a difference in cardiac tissue between the two ventricles [16].

We have previously shown that chronic hypoxia induces both cytoprotective and injury related transcriptomic reprogramming [6]. The protective program induces survival pathways and the deleterious program triggers cell death signaling [6]. Any imbalance between these two programs would result in either cell survival or death. Therefore, modulating the balance between these two programs offers the potential to develop strategies for cardioprotection. Our data suggest that, in cyanotic pediatric heart, the increase in Gab1 expression is part of the survival pathway. Additionally, the reduction of apoptosis observed following the overexpression of Gab1 suggests a critical and prosurvival role for Gab1 in rat neonatal cardiomyocytes. This is in line with previous reports describing an antiapoptotic function for Gab1 [12]. It has also been shown that SHP2 can help to promote cell survival by the activation of the Raf/MEK/ERK signalling pathway [17].

Gab1 has been shown to play an antiapoptotic role in oxidative condition [12]. It would be interesting to see how Gab1 expression levels would be following corrective surgery for cyanotic congenital heart disease. Similarly, it would be interesting to examine the removal of hypoxic stress in an experimental model. One can speculate that the removal of hypoxic stress would result in the return of Gab1 expression to normoxic condition levels. Additionally we would expect thatan abrupt reoxygenation could modulate the levels of this unique protein. Furthermore, the normal transition from foetal to neonatal circulation may affect Gab1 expression levels.

Gab1 expression changes following hypoxia may be controversial considering the observed difference between the* in vivo* and* in vitro* situations. However, it has previously been shown that oxygen availability can play a critical role in defining the cellular responses to stimuli [18]. Compared to the cell culture system, the* in vivo* situation adds another level of complexity. It is likely that other* in vivo* signalling pathways come into play that could result in a different response to hypoxia as compared to culture system. In the above-mentioned study, it has been shown that whereas IGF signaling promotes muscle cell differentiation under normoxia, it stimulates proliferation under hypoxia by differentially regulating multiple signaling pathways [18]. A possible mechanism involved in modulating Gab1 expression could be the HIF1 alpha-signaling pathway.

SHP2 and p85 expressions showed no difference between cyanotic and acyanotic TOF patients; however, they decreased, although it was not significant for p85, in hypoxic myocytes. A plausible explanation is the difference between RV tissue collected from TOF patients and the mixed cell population harvested from LV and RV of neonatal rats. There is evidence that cardiac tissue obtained from the two ventricles has different expression profile and can respond differently to stimuli [16].

Altogether, our data provide strong evidence that Gab1 is important for cardiomyocytes survival following hypoxic stress. Gab1 represents a potential target for cardioprotection.
